# Regional Disparities in Multiple Sclerosis Care in Poland: Epidemiology, Workforce Challenges, and Continuity of Care

**DOI:** 10.7759/cureus.92027

**Published:** 2025-09-10

**Authors:** Katarzyna Brukało

**Affiliations:** 1 Department of Health Policy, School of Public Health, Medical University of Silesia, Bytom, POL

**Keywords:** health policy, health services accessibility, health workforce, multiple sclerosis, regional disparities

## Abstract

Introduction

Multiple sclerosis (MS) is a chronic, immune-mediated disease of the central nervous system with a substantial socioeconomic burden. Despite universal healthcare coverage, disparities in access to specialized care and disease-modifying treatment persist in Poland. Understanding these disparities, particularly their regional dimension, is essential for optimizing workforce planning and improving continuity of care.

Methods

I conducted a retrospective, nationwide analysis of MS epidemiology, neurology workforce availability, treatment infrastructure, and patient care pathways in Poland using official health statistics from 2009 to 2023. Data included prevalence, incidence, number, and age structure of neurologists, distribution of first- and second-line treatment centers, patient migration rates, and waiting times between the first and second visit. Descriptive statistics and correlation analyses were performed at the regional level.

Results

Between 2019 and 2023, MS prevalence increased by 11.6% (from 61,693 to 68,850 cases), while incidence remained stable (+2.3%). The number of neurologists grew by only 3.4%, with over one-third at retirement age in several regions. Patient load per neurologist varied twofold across regions (11.6 in Mazowieckie vs. 21.0 in Opolskie), and higher workloads were associated with longer waiting times (r = 0.42) and higher patient migration rates (r = 0.39). The median waiting time between the first and second visit was 30 days, but exceeded 90 days for one in five patients. Regions with fewer second-line treatment centers and aging workforces showed the greatest continuity-of-care challenges.

Conclusions

Significant regional disparities in MS care exist in Poland, driven by uneven workforce distribution, an aging neurology workforce, and unequal access to specialized centers. Targeted interventions should prioritize high-burden, underserved regions, expand second-line treatment capacity, and address workforce aging to ensure equitable and timely care for MS patients.

## Introduction

Multiple sclerosis (MS) is a chronic, immune-mediated neurological disorder and one of the principal causes of non-traumatic disability among young adults worldwide. The disease is characterized by demyelination, axonal loss, and neurodegeneration within the central nervous system, leading to a wide spectrum of physical, cognitive, and psychosocial impairments [1,2]. In recent decades, the global burden of MS has increased substantially, with prevalence rates now exceeding 35 per 100,000 population in many countries and surpassing 100 per 100,000 in high-incidence regions [3,4]. This growth reflects both true increases in disease occurrence and improved survival, as well as enhanced diagnostic capabilities through the adoption of MRI and revised diagnostic criteria [2,5].

In Europe, the prevalence of MS varies widely between countries and even within national borders, reflecting differences in genetic susceptibility, environmental risk factors, and healthcare system organization [3,6]. Poland has historically been classified as a medium-to-high prevalence country for MS [4]. Earlier epidemiological studies reported regional prevalence rates of approximately 110-115 per 100,000 inhabitants [4], while a more recent nationwide analysis based on administrative health claims estimated a prevalence of 131.2 per 100,000 and incidence of 6.6 per 100,000 in 2019 [7]. However, these studies have predominantly focused on quantifying disease occurrence without exploring whether the increasing disease burden is matched by adequate healthcare resources or whether it leads to inequalities in access to diagnosis and treatment.

The introduction of disease-modifying therapies (DMTs) has transformed the prognosis of MS, with early initiation associated with reduced relapse rates and slower disability progression [5,8]. Nevertheless, access to these treatments depends on timely diagnosis, availability of specialized neurology services, and equitable distribution of treatment centers [8,9]. In Poland, treatment is delivered through a network of drug program centers, yet previous reports have highlighted significant regional variation in access to care and long-standing shortages in the neurology workforce [9,10]. Such disparities can result in delayed treatment initiation, higher rates of patient migration between regions, and increased strain on already overburdened facilities.

Understanding the relationship between MS epidemiology and healthcare system capacity is therefore essential for translating epidemiological data into targeted policy interventions. The primary objective of this study was to assess regional disparities in MS care in Poland by linking epidemiological indicators (prevalence and incidence) with neurology workforce capacity, treatment infrastructure, and continuity-of-care measures. The secondary objectives were (i) to identify voivodeships where high patient load, an aging workforce, and long waiting times converge; (ii) to examine associations between neurologist workload, patient migration, and waiting times as indicators of system efficiency.

For clarity, "first-line treatment centers" are defined as facilities authorized to initiate and monitor DMT, while "second-line centers" provide access to advanced therapies requiring additional authorization. Since 2013, two drug programs for patients with a confirmed diagnosis of MS have been available in Poland. First-line therapies include medications used to initiate treatment, while second-line therapies are applied in cases of insufficient response to first-line drugs or in patients with rapidly evolving severe disease. "Patient migration" refers to MS patients receiving treatment outside their voivodeship of residence. The main analyses focused on the years 2019-2023; waiting-time data were available only for 2018 and are presented as supplementary indicators of continuity of care. By combining descriptive epidemiology with health system indicators, this study establishes a model for data-driven healthcare planning and responsiveness.

## Materials and methods

Study design and data sources

This was a nationwide, cross-sectional analysis based on publicly available datasets from the Polish Ministry of Health’s System and Implementation Analysis Database Platform (Baza Analiz Systemowych i Wdrożeniowych - BASiW) portal, https://basiw.mz.gov.pl, accessed June 2025. The study covered data for the period 2009-2023, with specific time ranges depending on data availability for each indicator.

The following datasets were used:

(1) Epidemiological indicators - annual prevalence (chorobowość) and incidence (zapadalność) of MS by voivodeship (province) and year, 2009-2023. (2) Healthcare workforce - number of practicing neurologists and neurologists of retirement age (≥ 65 years) by voivodeship and year, 2009-2023. (3) Treatment infrastructure - number and distribution of first- and second-line treatment centers for MS by voivodeship and year. (4) Continuity of care indicators - waiting time (in days) between the first and second neurology visits for MS patients, by voivodeship (data for 2018). (5) Patient pathways - place of first, second, and third visit (primary health care, specialist outpatient clinic, hospital admission, or drug program center) for newly diagnosed MS patients. (6) Patient migration - percentage of patients treated outside their voivodeship of residence.

Data processing and definitions

For prevalence and incidence rates, population denominators by voivodeship were obtained from Statistics Poland (Główny Urząd Statystyczny, GUS). Rates were expressed per 10,000 inhabitants to allow regional comparison. The neurologist workload was calculated as the number of MS patients per practicing neurologist in a given voivodeship. The proportion of retirement-age neurologists was calculated relative to the total number of practicing neurologists in the region. Missing values were coded as “not available” and excluded from correlation analyses. For example, data on second-line treatment centers were not available for 2023 and were therefore omitted from these analyses.

"Patient migration" was defined as the proportion of MS patients receiving treatment outside their voivodeship of residence, calculated once per year. "Patients per neurologist" was calculated by dividing the number of MS patients in a given voivodeship and year by the total number of practicing neurologists (headcount, not full-time equivalents) in the same year. "Waiting time" was defined as the interval in days between the first and second recorded neurology visits for a given patient. Waiting-time data were available only for 2018 and were summarized for each voivodeship as median and interquartile range (IQR). "Patient pathways" were reconstructed by linking administrative records to identify the sequence of first, second, and subsequent neurology visits, classified as primary healthcare, specialist outpatient clinic, hospital admission, or drug program center. "Treatment centers" were classified into first-line (facilities authorized to initiate and monitor DMT) and second-line (facilities providing access to advanced therapies requiring additional authorization).

Statistical analysis

Descriptive statistics were used to present national trends and regional differences. Continuous variables were expressed as means, medians, and ranges; categorical variables as counts and percentages. Interregional differences were examined by comparing prevalence, incidence, workforce indicators, infrastructure availability, and patient pathway characteristics. Correlation analysis (Pearson’s r) was conducted to assess relationships between neurologist workload, retirement-age proportion, patient migration, and waiting time. No individual-level patient identifiers were used in this study; all data were aggregated and publicly available, and hence ethical approval was not required.

## Results

National trends (2019-2023)

Between 2019 and 2023, the number of patients with MS in Poland showed a consistent upward trend. The prevalence increased from 61,693 cases in 2019 to 68,850 in 2023, representing an 11.6% rise. During the same period, the incidence rate remained relatively stable, increasing only slightly from 3,004 to 3,073 new cases annually (+2.3%). However, the growth in the number of practicing neurologists did not keep pace with the increase in disease burden - their number rose from 4,677 to 4,834 (+3.4%) (Table 1)

**Table 1:** National trends in multiple sclerosis (MS) prevalence, incidence, and neurology workforce capacity in Poland, 2019–2023

Year	Prevalence (n)	Incidence (n)	Number of practicing neurologists	First-line treatment centers	Second-line treatment centers
2019	61,693	3,004	4,677	127	55
2020	63,121	2,409	4,649	127	61
2021	64,762	2,820	4,847	126	62
2022	66,740	3,053	4,739	125	68
2023	68,850	3,073	4,834	129	No data

In parallel, the network of treatment centers expanded. The number of first-line treatment centers increased from 66 in 2009 to 129 in 2023, and second-line centers from 29 in 2013 to 68 in 2022. Despite this growth, the expansion rate of infrastructure remained lower than the growth rate of the patient population, suggesting a widening gap between service demand and supply.

Regional profile (2023)

Data from 2023 revealed substantial interregional differences in both disease burden and workforce availability (Table 2).

**Table 2:** Regional distribution of multiple sclerosis (MS) prevalence, incidence, patient load per neurologist, and patient migration rates in Poland, 2023

Voivodeship	Prevalence (/10,000)	Prevalence (n)	Incidence (/10,000)	Patients per neurologist
Lower Silesia	19.43	5,594	0.78	19.16
Kuyavia-Pomerania	17.68	3,529	0.72	18.48
Lublin	21.01	4,225	0.94	14.23
Lubusz	15.35	1,497	0.59	20.79
Łódź	20.65	4,878	0.82	15.2
Lesser Poland	16.62	5,699	0.85	16.66
Masovia	18.32	10,097	0.92	11.63
Opole	16.85	1,578	0.61	21.04
Subcarpathia	22.27	4,614	0.72	17.75
Podlaskie	16.07	1,829	0.74	15.63
Pomerania	17.38	4,101	0.83	16.4
Silesia	20.29	8,765	1.01	15.16
Świętokrzyskie	17.3	2,021	0.81	15.31
Warmia-Masuria	15.36	2,086	0.61	16.43
Greater Poland	17.35	6,050	0.8	20.37
West Pomerania	14.02	2,287	0.52	17.2

The highest prevalence per 10,000 inhabitants was observed in Subcarpathia (22.27/10,000) and Lublin (21.01/10,000), while the lowest occurred in West Pomerania (14.02/10,000) and Lubusz (15.35/10,000). Incidence rates followed a different geographical distribution, with Silesia recording the highest value (1.01/10,000), followed by Lublin (0.94/10,000) and Masovia (0.92/10,000); the lowest incidence was seen in West Pomerania (0.52/10,000) and Lubusz (0.59/10,000).

The MS patient load per neurologist varied more than twofold between regions. In Masovia, there were 11.6 patients per neurologist, compared to 21.0 in Opole. High loads (>19 patients per neurologist) were also found in Lubusz (20.79), Greater Poland (20.37), and Lower Silesia (19.16). Patient migration - defined as receiving treatment outside the region of residence - was highest in Opole (19.6%), Łódź (18.7%), and Masovia (18.0%), and lowest in West Pomerania (6.3%) and Świętokrzyskie (8.9%).

Treatment centers were not evenly distributed. Regions with the highest patient migration rates tended to have fewer second-line treatment centers, with larger distances between them compared to central regions. An additional barrier to access was the high proportion of neurologists of retirement age, exceeding 35% in West Pomerania (38.5%), Łódź (38.3%), and Silesia (36.0%). The combination of a high patient load, an aging workforce, and limited infrastructure created particularly challenging conditions, exemplified by Opole, which had both the highest patient load per neurologist and the highest percentage of patients treated outside their region.

Workforce gap and age structure

The age structure of the neurology workforce in 2023 indicates a significant risk of further deterioration in service accessibility for MS patients. The proportion of neurologists of retirement age exceeded 35% in three regions: West Pomerania (38.5%), Łódź (38.3%), and Silesia (36.0%) (Table 3).

**Table 3:** Percentage of neurologists of retirement age, patient load per neurologist, and proportion of patients receiving treatment outside their region of residence in Poland, 2023

Voivodeship	Retirement age neurologists, %	Patient load per neurologist	Patient migration, %
West Pomerania	38.5	17.9	6.3
Łódź	38.3	18.7	18.7
Silesia	36	18.9	14.2
Lower Silesia	33.2	19.2	15.8
Warmia-Masuria	31.1	17	11.9
Opole	28.4	21	19.6
Masovia	29.8	11.6	18
Lubusz	35.4	19.5	19.2
Greater Poland	32.5	19.4	19.1
Subcarpathia	27.2	15.8	9.8
Kuyavia-Pomerania	28.1	16.5	12.3
Lublin	30.4	14.8	10.5
Pomerania	29	15.5	13.4
Podlaskie	27.8	14	10.1
Lesser Poland	26.9	13.9	9.5
Świętokrzyskie	25.5	13.5	8.9

In five other regions, this share was above 30%, including both high-burden regions (e.g., Lower Silesia - 33.2%) and regions with medium prevalence rates (e.g., Warmia-Masuria - 31.1%).

The situation was most critical in regions where a high proportion of retirement-age physicians coincided with a large patient load per neurologist and limited second-line treatment infrastructure. In Opole, despite a retirement-age share of “only” 28.4%, the highest patient load in the country (21.0 per neurologist) combined with high patient migration (19.6%) pointed to an already existing - and potentially deepening - workforce gap. Conversely, in Masovia, the patient load was the lowest in the country (11.6 per neurologist), yet the retirement-age share was 29.8%, suggesting that accessibility may deteriorate within a few years.

Continuity of care

Analysis of service accessibility throughout the MS patient care pathway indicated that waiting time between the first and second visit is a key factor affecting continuity of care. Nationwide in 2018, the median waiting time was 30 days (Table 4), with a markedly skewed distribution.

**Table 4:** Median waiting time between the first and second neurology visit for patients with multiple sclerosis in Poland, 2018, by region

Voivodeship	Median waiting time (days)
Lower Silesia	40
Kuyavia-Pomerania	35
Lublin	37
Lubusz	39
Łódź	42
Lesser Poland	32.5
Masovia	39
Opole	41
Subcarpathia	41.5
Podlaskie	39
Pomerania	42
Silesia	36
Świętokrzyskie	28
Warmia-Masuria	39
Greater Poland	38.5
West Pomerania	28

Over 40% of patients attended their second visit within 14 days of the first, but for approximately 20%, this interval exceeded 90 days. Extreme values reached as high as 365 days, pointing to rare but significant delays in treatment initiation. Waiting times varied regionally, with the longest intervals found in regions with the highest neurologist workload and limited numbers of treatment centers. For example, in Opole - with the highest workload - the median waiting time was 41 days, compared to 39 days in Masovia, where the workload was lowest.

**Figure 1:**
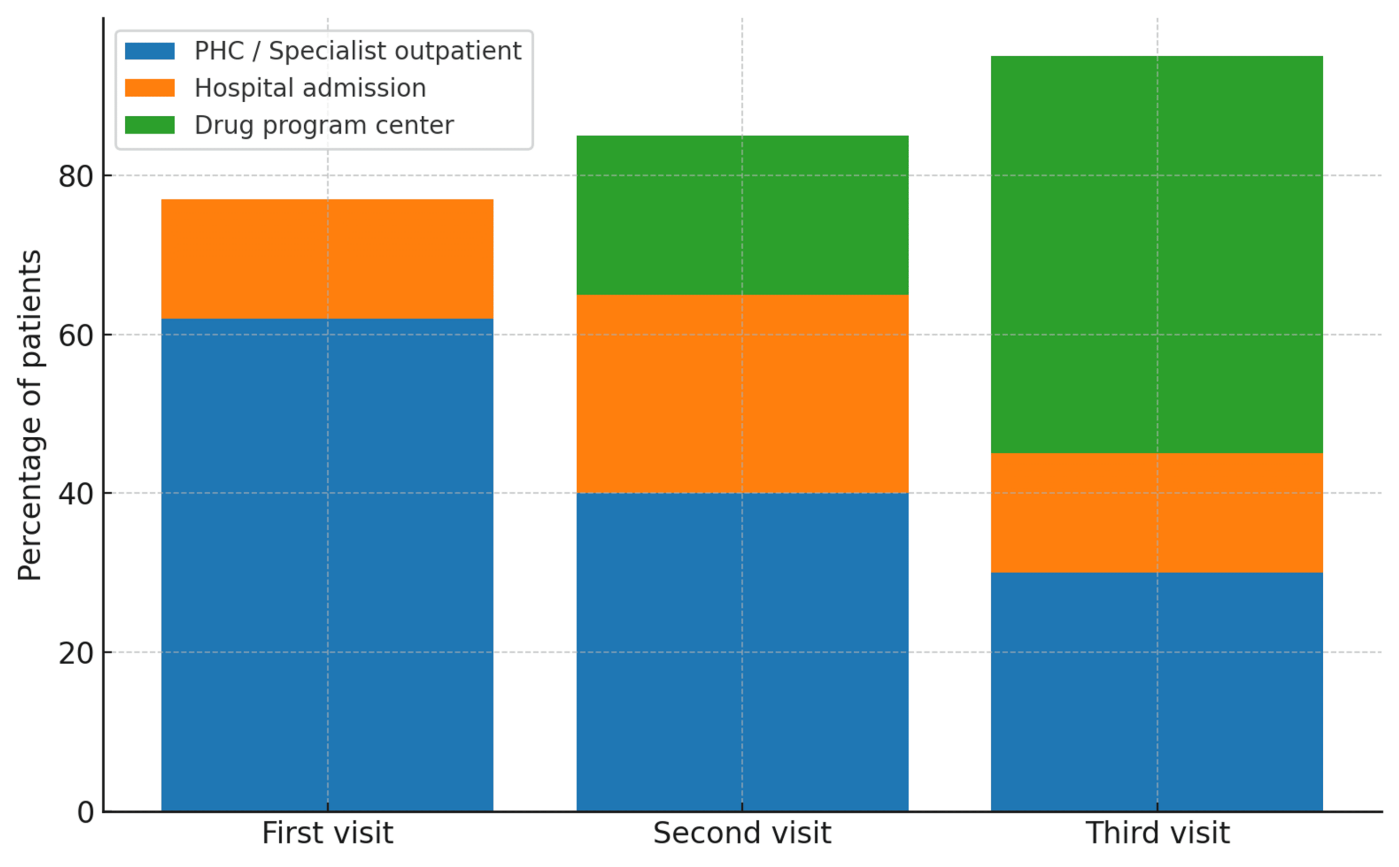
Patient pathways from the first to the third recorded healthcare visit for individuals with multiple sclerosis in Poland

Patient pathway data made it possible to identify typical treatment trajectories and potential bottlenecks. The first visit most often took place in primary health care or ambulatory specialist care (together over 60% of cases), followed by referral to a specialist hospital or a drug program center (Figure 1).

A notable proportion of patients (approx. 15%) had their first visit as a hospital admission, which may reflect acute symptom onset or the absence of prior outpatient diagnostics. Pathway analysis across subsequent visits revealed that in some cases, patients repeatedly returned to primary or specialist outpatient care before being enrolled in a drug program, potentially prolonging the time to initiation of disease-modifying treatment.

Relationships between indicators

Cross-analysis of the studied indicators revealed clear relationships between workforce workload, infrastructure availability, patient migration, and waiting time. Regions with high neurologist workloads tended to have both a greater proportion of patients treated outside their home region and longer waiting times between the first and second visits. For instance, in Opole, the workload was 21.0 patients per neurologist, patient migration reached 19.6%, and the median waiting time was 45 days.

**Figure 2:**
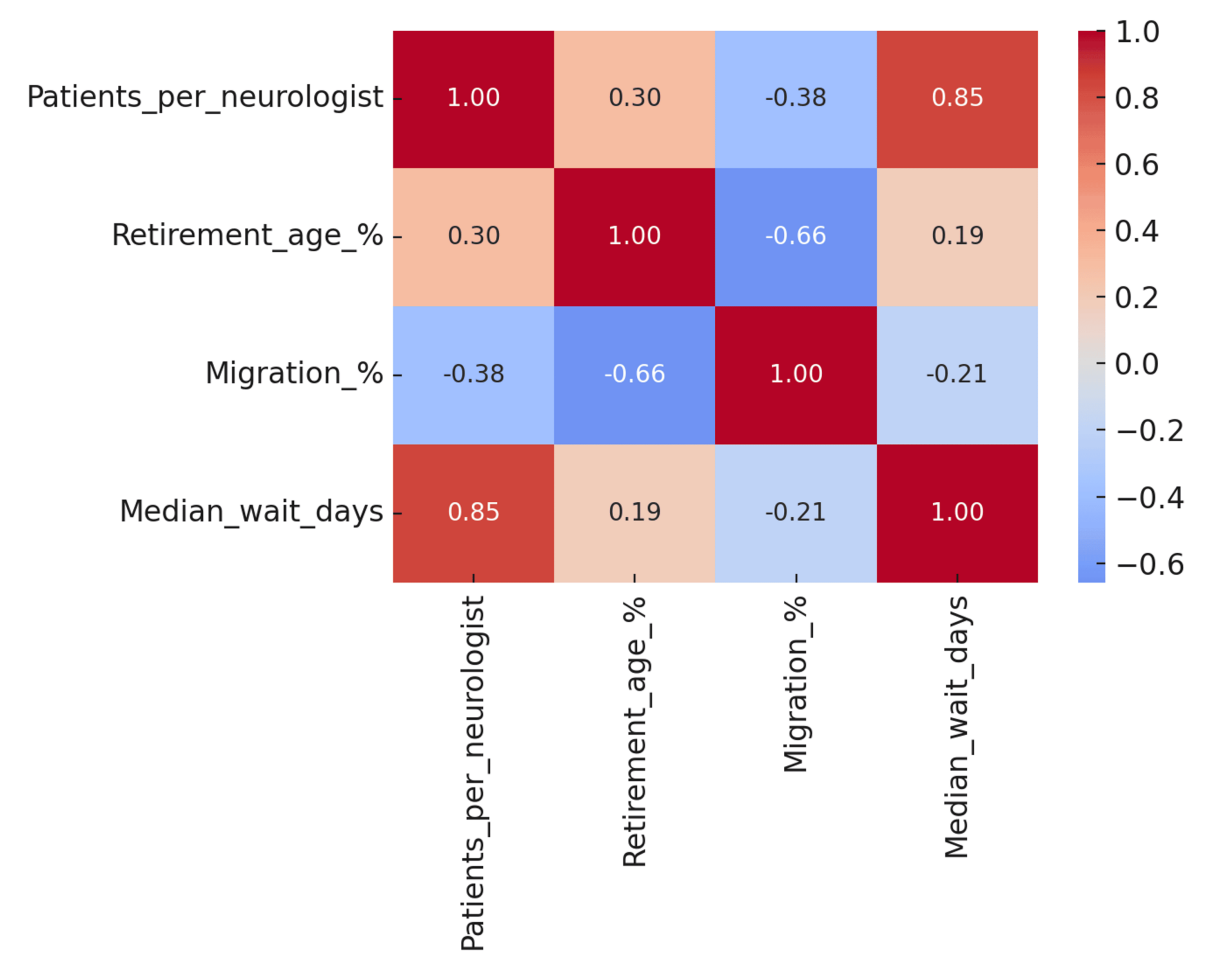
Relationships between neurologist workload, patient migration, and median waiting time between the first and second neurology visit in Polish regions

The opposite pattern was seen in regions with relatively low workloads, such as Masovia, where the migration rate was high (18.0%) but the waiting time was much shorter (21 days), suggesting that migration in this case may be driven more by patient preference for specialized centers than by lack of available appointments. Correlation analysis showed that the neurologist's workload was positively correlated with both patient migration (r = 0.42) and waiting time (r = 0.39) (Figure 2).

This suggests that workforce aging may be one of the factors exacerbating access problems, especially in regions already experiencing high workloads. These relationships underscore the need to address workforce policy, treatment center distribution, and patient pathway coordination as components of an integrated system, in which a change in one element affects the others.

## Discussion

This nationwide analysis reveals substantial and persistent regional disparities in both the burden of multiple sclerosis (MS) and the capacity of neurology services in Poland. Between 2019 and 2023, the prevalence of MS increased by over 11%, while the number of practicing neurologists grew by only 3.4%. This imbalance mirrors trends observed in other European countries, where rising prevalence and incidence of MS have not been matched by proportional workforce growth, leading to concerns about timely access to diagnosis and treatment [4,11].

The patient-to-neurologist ratio varied more than twofold between voivodeships, from 11.6 in Masovia to 21.0 in Opole. Regions with ratios exceeding 18:1 - Opole, Lubusz, Greater Poland, and Lower Silesia - are particularly vulnerable to service strain and should be prioritized for targeted workforce reinforcement through dedicated contracts or relocation incentives coordinated at the national level [12]. Workforce age structure adds another layer of risk. In West Pomerania, Łódź, and Silesia, more than one-third of neurologists are already of retirement age, a finding consistent with the broader aging trend in the European neurology workforce [5,13]. Without proactive measures, these regions may face abrupt declines in capacity. Introducing flexible part-time roles, structured mentorship for junior neurologists, and teleconsultation support could help retain experienced professionals while ensuring knowledge transfer [14].

Infrastructure development has progressed, with increases in both first- and second-line treatment centers, yet distribution remains uneven. Regions with the highest migration rates-Opole, Łódź, and Masovia-often lack sufficient second-line centers. Establishing at least one additional facility in each of these regions, strategically located to minimize travel time, could reduce the need for patients to seek care outside their home voivodeship and address geographical inequities [15]. Continuity-of-care analysis highlights delays between the first and second neurology visits as a critical bottleneck. Although the national median interval was 30 days, in Opole it reached 45 days, and in rare cases exceeded three months. Such delays are clinically relevant, as early initiation of disease-modifying therapy is associated with better long-term outcomes [6,12]. Implementing a fast-track diagnostic referral protocol - requiring that the second visit for suspected MS be scheduled within 14 days - may help address this gap [13].

Finally, the absence of an integrated monitoring framework hampers timely responses to emerging service gaps. Linking BASiW epidemiological data with National Health Fund scheduling and prescription data could enable real-time tracking of prevalence, workforce capacity, waiting times, and treatment initiation by voivodeship. Such a system may support proactive policy adjustments and resource allocation before access problems become critical [11,14].

This study has several limitations. First, reliance on administrative data may introduce inaccuracies due to diagnostic coding errors and incomplete registry updates, which could lead to under- or overestimation of true disease burden. Second, the cross-sectional design precludes causal inference and restricts interpretation to associations. Third, temporal misalignment of indicators may bias comparisons: waiting-time data were available only for 2018, whereas epidemiological and workforce indicators extended to 2023. Fourth, workforce metrics were based on headcount rather than full-time equivalents or MS-specialist neurologists, and the ≥65 age threshold represents only a proxy for retirement risk rather than actual workload reduction. Fifth, the analysis did not apply age-standardization, which may limit comparability across voivodeships with different population structures. Finally, as an ecological study, the analysis does not account for potential regional-level confounders such as urbanicity, socioeconomic status, or referral patterns.

By combining epidemiological, workforce, and patient pathway data, this study provides an actionable framework for health system strengthening. The findings indicate that region-specific interventions, rather than uniform national measures, are essential to align service capacity with the geographic distribution of MS burden and to ensure equitable access to timely diagnosis and treatment.

## Conclusions

This nationwide assessment of MS care in Poland shows that the rising burden of disease is accompanied by uneven workforce capacity, infrastructure distribution, and service accessibility. By examining patient-to-neurologist ratios, age structure of the workforce, treatment center availability, waiting times, and patient migration patterns, this study identifies regions where these challenges converge and pose the greatest risk to care continuity. The results highlight that even within a single national healthcare system, access to timely and specialized MS care can vary substantially depending on place of residence. Improving equity in MS care may require targeted, region-specific interventions rather than uniform national measures. Priorities should include redistributing workforce capacity to high-burden regions, retaining experienced specialists through flexible and supportive employment models, expanding second-line treatment infrastructure in underserved areas, and streamlining diagnostic pathways to reduce waiting times. Developing an integrated monitoring system that links epidemiological, workforce, and service access indicators would provide the foundation for more responsive, data-driven policy decisions. These strategies not only address current inequities but also may help create a framework for improving care for other chronic neurological conditions facing similar systemic challenges.
